# The Ubiquitin E3 Ligase PRU2 Modulates Phosphate Uptake in Arabidopsis

**DOI:** 10.3390/ijms23042273

**Published:** 2022-02-18

**Authors:** Mi-Mi Sun, Yan Tian, Mei Chun, Yi-Fang Chen

**Affiliations:** State Key Laboratory of Plant Physiology and Biochemistry, College of Biological Sciences, China Agricultural University, Beijing 100193, China; sunmimi@cau.edu.cn (M.-M.S.); tianyan@cau.edu.cn (Y.T.); chunmei@cau.edu.cn (M.C.)

**Keywords:** low-Pi stress, ubiquitin E3 ligase, Pi acquisition

## Abstract

Phosphorus is an essential macronutrient for plants. The phosphate (Pi) concentration in soil solutions is typically low, and plants always suffer from low-Pi stress. During Pi starvation, a number of adaptive mechanisms in plants have evolved to increase Pi uptake, whereas the mechanisms are not very clear. Here, we report that an ubiquitin E3 ligase, PRU2, modulates Pi acquisition in Arabidopsis response to the low-Pi stress. The mutant *pru2* showed arsenate-resistant phenotypes and reduced Pi content and Pi uptake rate. The complementation with *PRU2* restored these to wild-type plants. PRU2 functioned as an ubiquitin E3 ligase, and the protein accumulation of PRU2 was elevated during Pi starvation. PRU2 interacted with a kinase CK2α1 and a ribosomal protein RPL10 and degraded CK2α1 and RPL10 under low-Pi stress. The in vitro phosphorylation assay showed that CK2α1 phosphorylated PHT1;1 at Ser-514, and prior reports demonstrated that the phosphorylation of PHT1;1 Ser-514 resulted in PHT1;1 retention in the endoplasmic reticulum. Then, the degradation of CK2α1 by PRU2 under low-Pi stress facilitated PHT1;1 to move to the plasma membrane to increase Arabidopsis Pi uptake. Taken together, this study demonstrated that the ubiquitin E3 ligase—PRU2—was an important positive regulator in modulating Pi acquisition in Arabidopsis response to low-Pi stress.

## 1. Introduction

Phosphorus (P) is an essential macronutrient for plant growth and development. P is a fundamental element of vital organic molecules, such as DNA, RNA, ATP, and membrane phospholipids. Whereas P is one of the least accessible macronutrients, the inorganic phosphate (Pi) concentration in soil solutions is typically 10 µM or less [1], which results in Pi starvation and impacts plant growth and survival. As a consequence, Pi shortage results in yield losses ranging from 25% to 60% in crops [2,3,4,5].

To cope with Pi limitations in the environment, plants have evolved by a range of morphological and physiological strategies to improve Pi acquisition. During Pi starvation, plants modulate their root system architectures (RSAs), such as repressing primary root growth, increasing lateral root formation and growth, and promoting root hair formation and elongation, to explore soil layers to forage for Pi, and some plant species form a mycorrhizal symbiosis structure with fungi to create an extended “root” system [5,6]. Under low-Pi (LP) stress, some plant species exude organic acids and acid phosphatases to solubilize P that is fixed in soils [7,8].

Plants absorb Pi from soils mainly via proton-coupled H_2_PO_4_^−^ phosphate transporter 1 (PHT1) subfamily proteins. There are nine and thirteen *PHT1* genes in Arabidopsis and rice, respectively [9,10,11,12]. Five of nine Arabidopsis PHT1 (AtPHT1;1/4/5/8/9) and nine of thirteen rice PHT1 (OsPT1/2/4/6/8/9/10/11/13) are involved in Pi uptake [11,12]. Most *PHT1* genes are expressed in roots and transcriptionally up-regulated during Pi starvation [9,10,11,12,13].

The PHT1 transporters can be regulated at a post-translational level to adapt to environmental Pi variability. The trafficking of PHT1 proteins from the endoplasmic reticulum (ER) to the plasma membrane (PM) is crucial for their Pi uptake function. PHOSPHATE TRANSPORTER TRAFFIC FACILITATOR (PHF) proteins, AtPHF1, and OsPHF1 facilitate PHT1 transporters trafficking from the ER to the PM [14,15,16,17]. The ER exit of Arabidopsis PHT1;1 is dependent on the phosphorylation status of the Ser-514 residue of AtPHT1;1, and the phosphorylation of AtPHT1;1 Ser-514 prevents its exit from the ER [15]. A further study revealed that under Pi-sufficient conditions, a rice casein kinase 2 (CK2) kinase holoenzyme, CK2α3/β3, phosphorylates OsPT8 at Ser-517, a conserved residue of AtPHT1;1 Ser-514, and the phosphorylated OsPT8^S517^ is retained in the ER [17]. Recently, Yang et al. [18] found that a rice protein phosphatase type 2C (PP2C) protein phosphatase, OsPP95, dephosphorylates OsPT8 at Ser-517 and promotes OsPT8 trafficking from the ER to the PM.

In this study, we obtained a mutant, named *phosphate response ubiquitin E3 ligase 2* (*pru2*), which was defective in Arabidopsis Pi acquisition. *PRU2*, also named *AtATL78*, encoded an ubiquitin E3 ligase, and protein accumulation of PRU2 was increased during Pi starvation, suggesting that PRU2 was a LP-response ubiquitin E3 ligase. Further molecular and biochemical data revealed that PRU2 interacted with CK2 kinases CK2α1 and CK2α2, and modulated the degradation of CK2α1 under LP stress. The in vitro phosphorylation assay showed that CK2α1 phosphorylated PHT1;1 at Ser-514. As the phosphorylation of PHT1;1 at Ser-514 prevented PHT1;1 exit from the ER [15], the degradation of CK2α1 by PRU2 facilitated PHT1;1 to traffic from the ER to the PM, thereby increasing Arabidopsis Pi uptake. This work contributed to a better understanding of plant Pi acquisition in response to LP stress.

## 2. Results

### 2.1. The pru2 Mutant Was Defective in Pi Uptake

**Figure 1:**
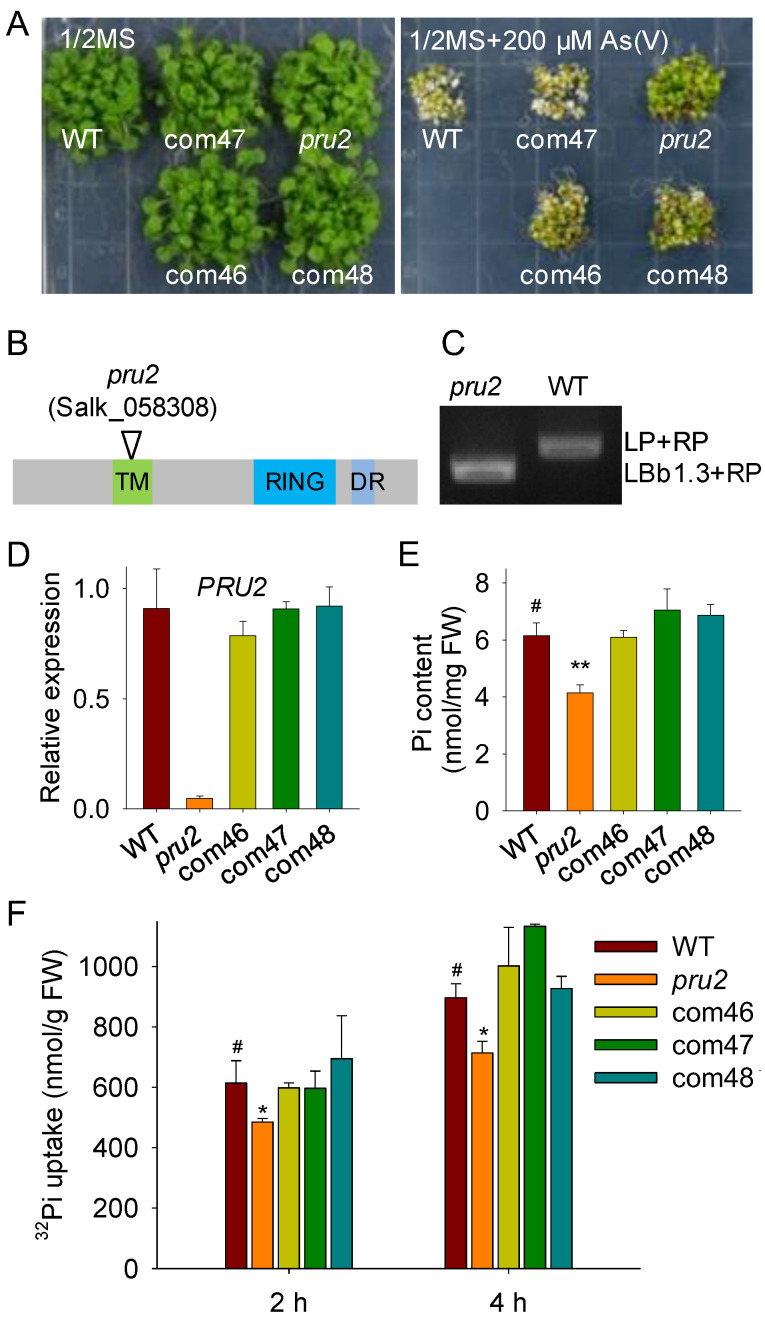
The *pru2* mutant was Arsenate (As(V))-resistant and defective in Pi uptake. (**A**) Phenotype comparison of the *pru2* mutant, complementation lines, and wild-type (WT) plants under As(V) stress. All genotypes were germinated and grown on a 1/2 Murashige and Skoog (MS) medium with or without 200 μM As(V) for 15 d. (**B**) Diagram of PRU2 showing the insertion site of *pru2* mutant. (**C**) Genotyping PCR analysis of the *pru2* mutant. (**D**) RT-qPCR analysis of *PRU2* in the *pru2* mutant, complementation lines, and WT plants. Data are shown as mean ± Standard Error (SE) (*n* = 3). (**E**) Pi content measurement. All genotypes germinated and grown on an MS medium for 7 d and then transferred to a low-Pi (LP) medium for 5 d. Data are shown as mean ± SE (*n* = 3). (**F**) ^32^Pi uptake capacity measured in 7-day-old seedlings grown on an LP medium for 3 d. Data are shown as mean ± SE (*n* = 3). Asterisks indicate significant differences compared with WT plants (^#^) by Student’s *t* test. *, *p*< 0.05; **, *p*< 0.01.

Arsenate (As(V)) is nonessential and toxic to plants, and it enters into plant cells mainly via Pi transporters [19,20,21,22]. In order to screen an E3 ligase that was involved in Arabidopsis Pi uptake, we screened 430 *e3* mutants [23], which had a T-DNA insertion in a putative E3 ligase under As(V) conditions. A mutant, named *pru2*, showed no obvious differences with wild-type plants when grown on a 1/2 Murashige and Skoog (MS) medium without As(V), whereas when grown on a 1/2 MS medium with 200 µM As(V), the *pru2* mutant displayed As(V)-tolerant phenotypes, with green and larger leaves, compared with wild-type plants (Figure 1A). The As(V)-tolerant phenotypes of the *pru2* mutant were confirmed with seeds from two independent harvests.

The *pru2* mutant was Salk_058308C, which had a T-DNA insertion in AT1G49230 (Figure 1B). Then, we designated the affected gene *PRU2* (*PHOSPHATE RESPONSE UBIQUITIN E3 LIGASE 2*, AT1G49230). AT1G49230 was also named *ARABIDOPSIS TÓXICOS EN LEVADURA 78* (*ATL78*). The *pru2* mutant was a homozygous T-DNA insertion mutant of *PRU2*/AT1G49230 (Figure 1C), and the transcript level of *PRU2* was significantly repressed in the *pru2* mutant (Figure 1D).

To confirm that the As(V) tolerance of the *pru2* mutant was due to the T-DNA insertion in *PRU2*, we generated complementation lines. The *proPRU2:PRU2–GFP* *(Green* *Fluorescent* *Protein)* was constructed and transformed into the *pru2* mutant to generate *pru2* complementation lines. Three complementation lines (com46, com47, and com48), which displayed similar transcript levels of *PRU2* as wild-type plants (Figure 1D), were selected for further assays. These three complementation lines showed no obvious differences with wild-type plants when grown on a 1/2 MS medium and rescued the As(V) tolerance of the *pru2* mutant (Figure 1A), suggesting that the disruption of *PRU2* enhanced Arabidopsis As(V) resistance.

Since As(V) entry into plant cells is mainly via Pi transporters [19,20,22], we hypothesized that PRU2 modulated Arabidopsis Pi acquisition. The Pi contents of various plants were first measured. These genotypes were germinated and grown on an MS medium for 7 d and then transferred to an LP medium for 5 d. The *pru2* mutant had a lower Pi content compared with the wild-type plants, and the Pi contents of three complementation lines were similar to those of the wild-type plants (Figure 1E). Further, Pi uptake rate was analyzed. Seven-day-old seedlings were grown on an LP medium for 3 d and then transferred into a Pi-uptake solution containing 500 µM Pi supplemented with ^32^P KH_2_PO_4_, and Pi uptake over a 4 h period was measured. The *pru2* mutant displayed a reduction in Pi uptake capacity compared to the wild-type plants, and three complementation lines rescued this Pi uptake defect of the *pru2* mutant (Figure 1F). Together, these data suggested that PRU2 positively modulated Arabidopsis Pi uptake.

### 2.2. RSA and Organic Acids Exudation in the pru2 Mutant under LP Stress

**Figure 2:**
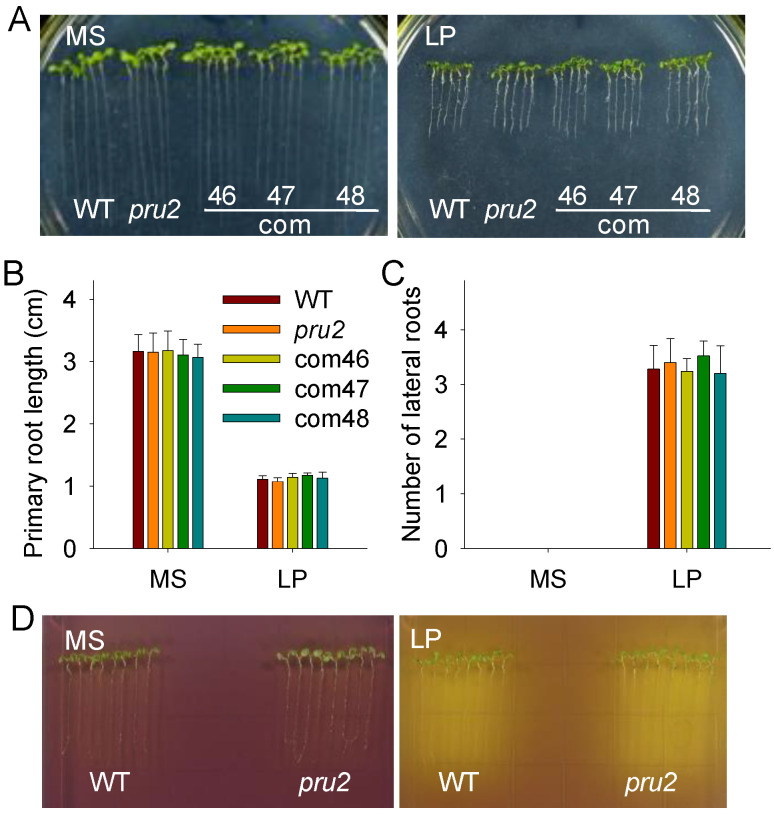
Phenotypic comparison of the *pru2* mutant under LP stress. (**A**) Phenotypic comparison among the *pru2* mutant, the complementation lines, and the WT seedlings germinated and grown under either an MS or an LP medium for 9 d. The primary root length (**B**) and the lateral root number (**C**) were measured in all genotypes treated as described in (**A**). Data are shown as mean ± SE (*n* = 25) of each genotype per treatment. (**D**) Visualization of the medium acidification around roots using a pH-sensitive dye bromocresol purple. Seven-day-old seedlings were transferred to an MS or LP medium containing this dye for 1 d.

The modulation of the RSA is an efficient adaptive response to Pi starvation in Arabidopsis [5,24,25]. Since the *pru2* mutant showed a reduced ^32^Pi uptake (Figure 1F), we wondered whether PRU2 had an effect on the RSA. The *pru2* mutant, complementation lines, and wild-type plants were germinated and grown in vertically oriented plates with an MS or LP medium for nine days. The Pi deficiency resulted in a significant reduction of primary root growth and an increase of lateral root numbers in the *pru2* mutant, the complementation lines, and the wild-type plants; however, the *pru2* mutant showed no obvious differences with the complementation lines and the wild-type plants (Figure 2A–C), suggesting that the Pi-deficiency-induced RSA was not modulated by PRU2.

An increase in the productions of extracellular organic acids and protons is a universal response of plants to Pi deficiency. PHT1;1 and PHT1;4 are the main Pi transporters for Arabidopsis Pi acquisition from environments [19], and both PHT1;1 and PHT1;4 are H^+^/H_2_PO_4_^−^ symporters. The exudative organic acids and protons facilitate Pi to be released from unavailable soil P forms and increase plant Pi uptake ability. A comparative analysis of organic acid and proton exudations was performed using a pH-sensitive dye bromocresol purple [26]. Pi deprivation significantly induced organic acid and proton exudation (Figure 2D), similar to those in previous reports [27,28]. However, no significant variation was observed between the *pru2* mutant and the wild-type plants (Figure 2D).

### 2.3. PRU2 Was a LP-Response Ubiquitin E3 Ligase

**Figure 3:**
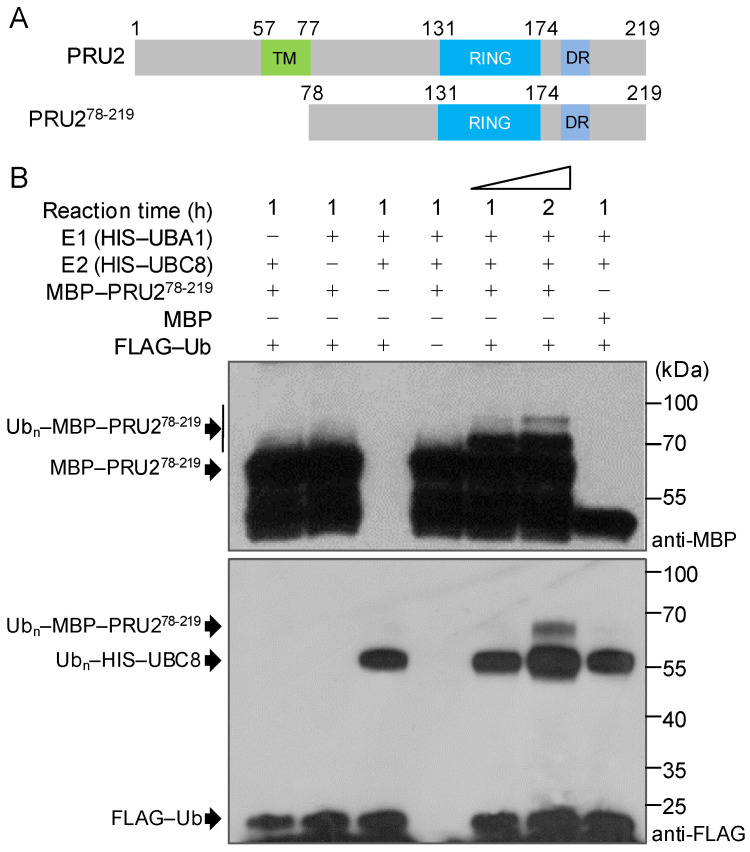
PRU2 had a self-ubiquitination activity in vitro. (**A**) Diagrams of the full-length and truncated PRU2. The numbers refer to the positions of the first or last amino acid in the conserved domains. TM, transmembrane; DR, diverse region. (**B**) In vitro self-ubiquitination assay. Maltose-Binding Protein (MBP)–PRU2^78-219^ fusion proteins (500 ng) were incubated at 30 °C for 1 or 2 h with or without ubiquitin (Ub), ubiquitin-activating enzyme (E1) (HIS–UBA1), and ubiquitin-conjugating enzyme (E2) (HIS–UBC8). Reaction products were analyzed by immunoblotting using an anti-MBP antibody or anti-FLAG antibody.

PRU2 had 219 amino acid residues and contained a putative transmembrane (TM) domain, a single C_3_H_2_C_3_-type RING motif (RING), and a diverse region (DR) (Figure 3A). To test the function of PRU2, we constructed *Maltose-Binding Protein* *(MBP)–PRU2*, *Glutathione S-Transferase* *(GST)–PRU2*, *HIS–PRU2*, and *CKS–PRU2–HIS* and expressed them in *Escherichia coli* (*E. coli*). Unfortunately, all these tagged PRU2 proteins were not soluble in the *E. coli*. Then, we generated the truncated PRU2, MBP–PRU2^78-219^, in which the TM of PRU2 was removed (Figure 3A) [29]. Further, the E3 ligase activity of MBP–PRU2^78-219^ was tested. Similar to a previous report [29], the truncated PRU2, PRU2^78-219^, showed an in vitro self-ubiquitination activity in the presence of ubiquitin-activating enzyme (E1), ubiquitin-conjugating enzyme (E2) (ubiquitin conjugating enzyme 8, UBC8), and ubiquitin (Ub) (Figure 3B). In addition, the polyubiquitination signal of PRU2^78-219^ was enhanced with the increase of the reaction time (Figure 3B). These data suggested that the truncated PRU2^78-219^ had an ubiquitin E3 ligase activity, and the fragment from 78 to 219 amino acids was important for the function of PRU2 as an ubiquitin E3 ligase.

**Figure 4:**
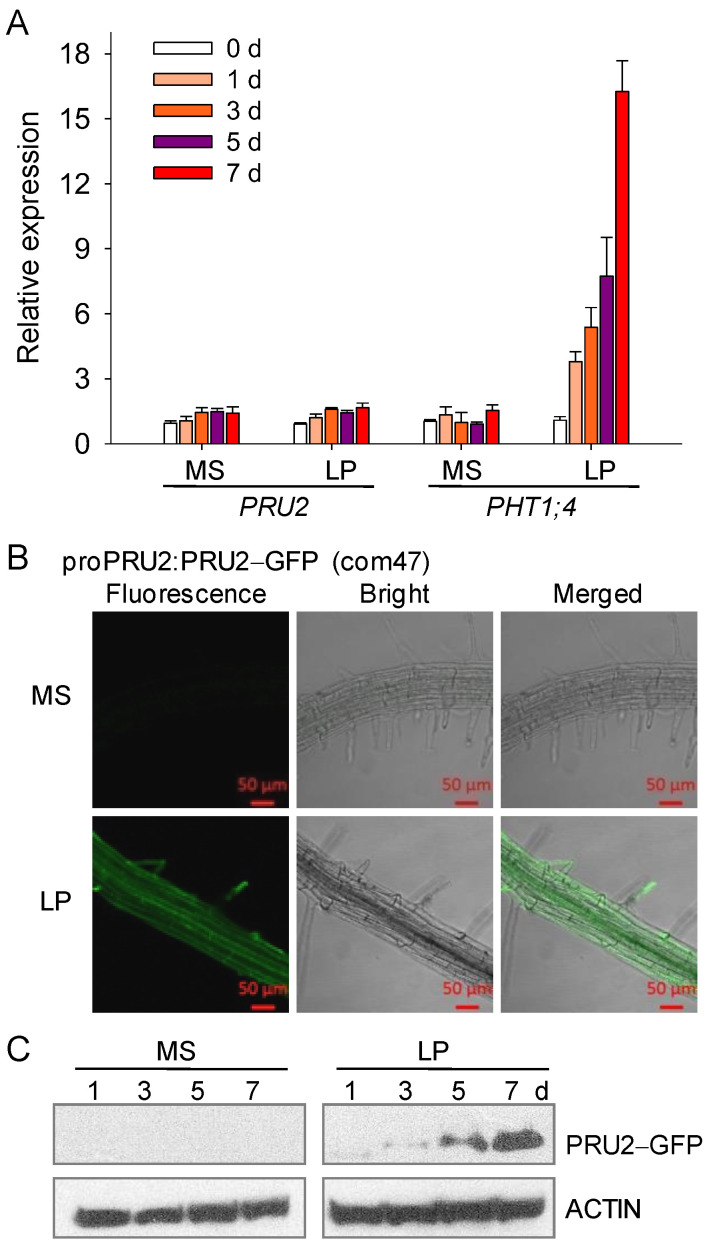
Transcript and protein accumulations of PRU2 under LP stress. (**A**) RT-qPCR analysis of *PRU2* and *PHT1;4* during Pi starvation. Seven-day-old WT seedlings were transferred to an LP medium, and then, the roots were harvested at the indicated time points for RNA extraction. Data are shown as mean ± SE (*n* = 3). (**B**) Fluorescence observation in the roots of the *proPRU2:PRU2–GFP* transgenic line (com47). The seven-day-old com47 was transferred to an MS or LP medium for 5 d. (**C**) Immunoblot analysis. The seven-day-old com47 was transferred to an MS or LP medium and then harvested at the indicated time points for immunoblot analysis. ACTIN was used as the loading control.

To further elucidate the role of PRU2, the transcript abundance of *PRU2* was measured during Pi starvation. Seven-day-old wild-type seedlings were transferred to an MS or LP medium, and then, the roots were harvested at the indicated time points. Similar to a previous report [19], the transcript level of *PHT1;4* was significantly increased during Pi starvation (Figure 4A),whereas the expression of *PRU2* was almost not changed LP stress (Figure 4A), indicating that PRU2 did not response to LP stress at a transcriptional level.

Next, we investigated the protein accumulation of PRU2 during Pi starvation. The complementation line, com47, could rescue the defects of the *pru2* mutant (Figure 1), indicating that the PRU2–GFP protein had a normal function as PRU2. Then, the GFP fluorescence of the PRU2–GFP in com47 was observed under Pi-sufficient or Pi-deficient conditions. The com47 was germinated and grown on an MS medium for seven days and then transferred to an MS or LP medium for five days. When grown under Pi-sufficient conditions (MS), the GFP fluorescence signals were very weak in com47 roots (Figure 4B). However, when grown under Pi-deficient conditions (LP), strong PRU2–GFP fluorescence signals were observed in the epidermis, cortex, vascular tissue, and root hair of com47 roots (Figure 4B), suggesting that the protein accumulation of PRU2 was elevated under LP stress. The PRU2 was expressed in the epidermis, cortexes, and vascular bundle in roots (Figure 4B). In cells, PRU2 was localized to the PM (Figure 4B) [29] and in the cytoplasm, typically in root hairs (Figure 4B). The expression patterns of PRU2 were consistent with its function in modulating Pi acquisition.

The protein accumulation of PRU2 during Pi starvation was further analyzed by immunoblotting. The seven-day-old com47 was transferred to an MS or LP medium and then harvested at the indicated time points. The PRU2–GFP protein was analyzed by immunoblotting using an anti-GFP antibody. The PRU2–GFP protein signaling was almost not detected in the com47 grown on an MS medium, whereas the PRU2–GFP proteins were gradually accumulated in the com47 during Pi starvation (Figure 4C), indicating that PRU2 was modulated at the post-translational level under LP stress. Together, our data demonstrated that PRU2 was a LP-response ubiquitin E3 ligase.

### 2.4. Identification Targets of PRU2

For PRU2 functioning as an ubiquitin E3 ligase, we then asked what were the targets of PRU2 under LP stress. First, we constructed an LP yeast library using seven-day-old Arabidopsis seedlings treated with LP stress for 1, 3, 5, and 7 d. We screened the LP yeast library with a full-length *PRU2* using the yeast two-hybrid method and obtained eight putative target genes (Table 1).

**Figure 5:**
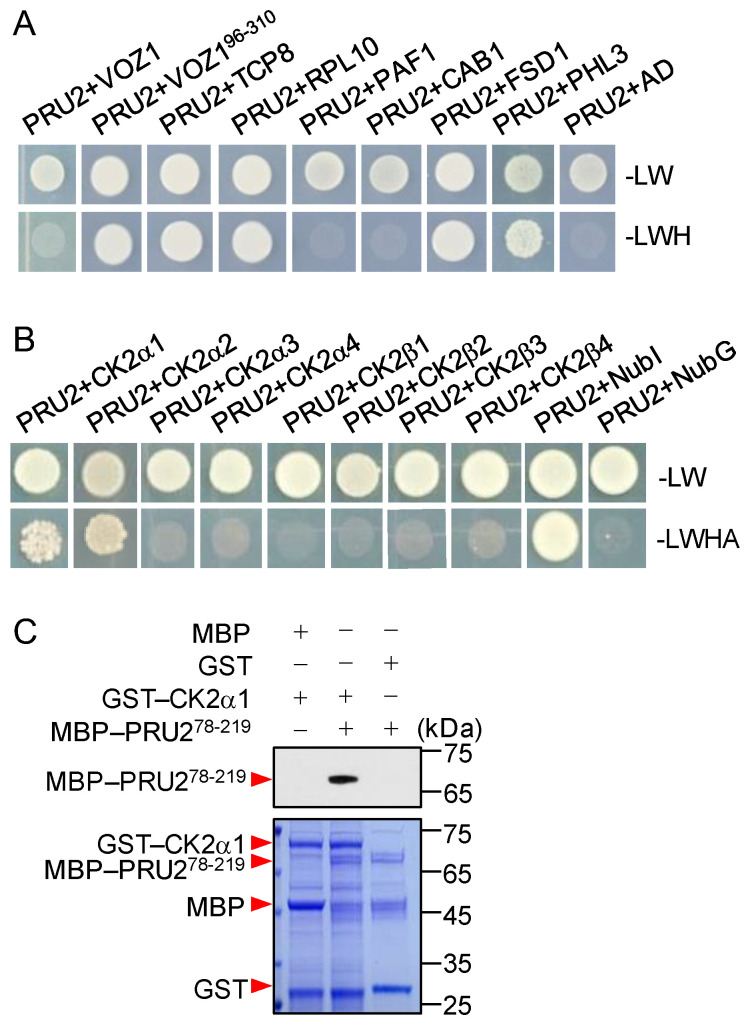
Interaction of PRU2 with its putative targets. (**A**,**B**) Yeast two-hybrid assay test results for an interaction between PRU2 and its putative targets. (**C**) In vitro pull-down assay between the recombinant proteins GST–CK2α1 and MBP–PRU2^78-219^. MBP–PRU2^78-219^ or MBP proteins were incubated with immobilized GST–CK2α1 or GST, and immunoprecipitated fractions were probed with an anti-MBP antibody.

The full-length coding sequences of these genes were cloned, and the interactions of the putative targets with PRU2 were separately tested in yeast. Five full-length proteins, TCP8, RPL10, FSD1, PHL3, and CK2α1, interacted with PRU2 in yeast (Figure 5A,B). The truncated VOZ1, VOZ1^96-310^, but not full-length VOZ1, could interact with PRU2 in yeast (Figure 5A).

A previous report demonstrated that rice CK2 kinase regulates the trafficking of OsPT8 during Pi starvation [17]. Rice CK2α3/β3 holoenzyme phosphorylates OsPT8 under Pi-sufficient conditions and inhibits OsPT8 from exiting from the ER to the PM [17]. There are four *CK2α* genes and four *CK2β* genes in the Arabidopsis genome [30]. We hypothesized that other CK2 subunits interacted with PRU2. Then, Arabidopsis *CK2α* and *CK2β* genes were cloned. Two CK2 subunits, CK2α1 and CK2α2, interacted with PRU2 in yeast (Figure 5B). The interaction was further tested using a pull-down assay. The GST–CK2α1 and GST–CK2α2 fusion constructs were expressed in *E. coli* separately. We obtained a soluble full-length GST–CK2α1 protein in *E. coli*, but not enough GST–CK2α2. The pull-down assay showed that GST–CK2α1 interacted with MBP–PRU2^78-219^ in vitro (Figure 5C). These data also indicated that the fragment from 78 to 219 amino acids of PRU2 was important for PRU2 to recognize its targets.

### 2.5. RPL10 and CK2α1 Were Targets of PRU2 under LP Stress

**Figure 6:**
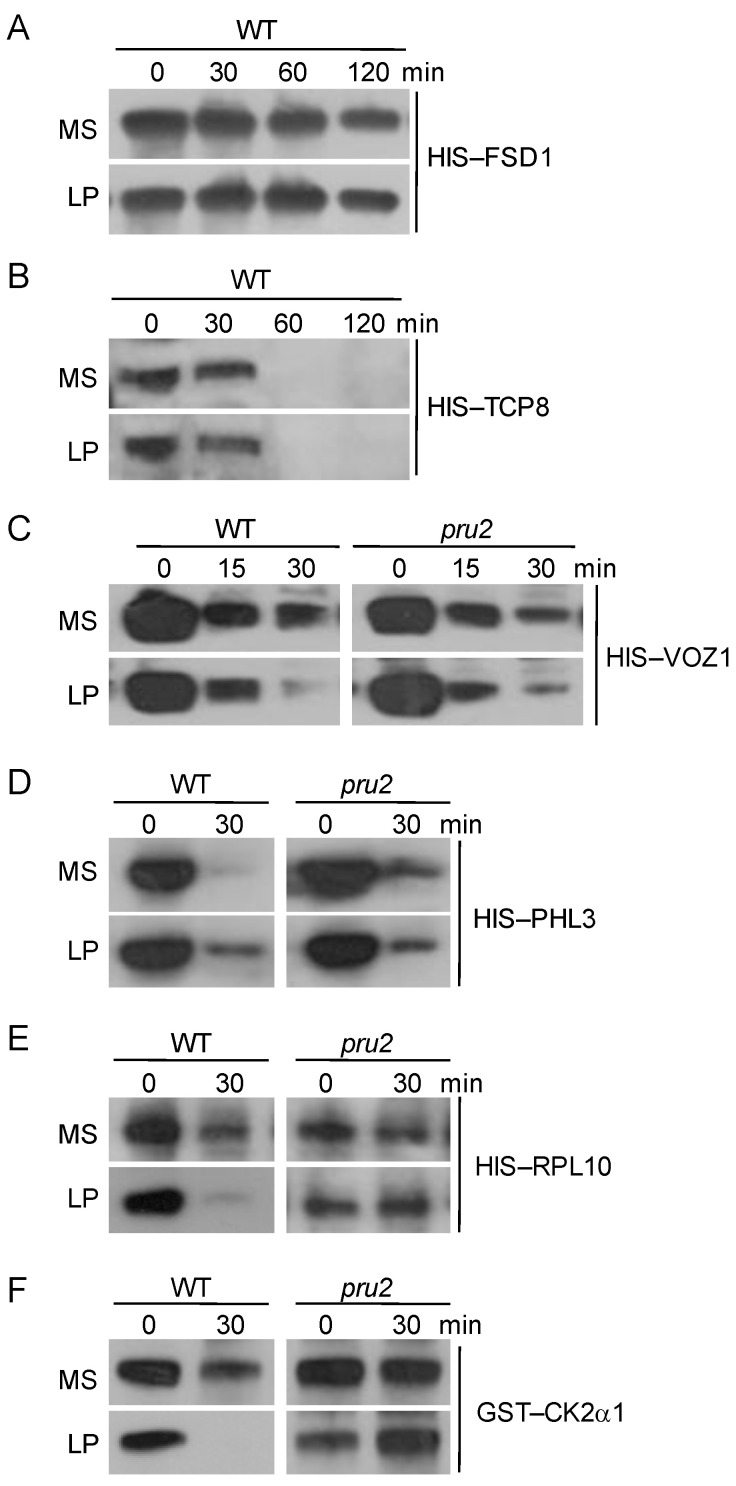
Cell-free degradation assay of PRU2 targets during Pi starvation. The seven-day-old *pru2* mutant and the WT seedlings were transferred to an MS or LP medium for 3 d and then harvested for the protein extraction. The protein extracts were incubated with the recombinant fusion proteins, i.e., HIS–FSD1 (**A**), HIS–TCP8 (**B**), HIS–VOZ1 (**C**), HIS–PHL3 (**D**), HIS–RPL10 (**E**), and GST–CK2α1 (**F**) separately, and the time-dependent changes of the protein levels were monitored by immunoblotting with an anti-HIS antibody or anti-GST antibody.

For PRU2 functioning as an ubiquitin E3 ligase (Figure 3) and the elevation of the protein accumulation of PRU2 during Pi starvation (Figure 4), we then wondered which interaction protein was the target of PRU2 under LP stress. The constructs of *HIS–VOZ1*, *HIS–TCP8*, *HIS–RPL10*, *HIS–FSD1*, *HIS–PHL3*, and *GST–CK2α1* were generated, and the recombinant fusion proteins were expressed and purified from *E. coli*. Then, a cell-free degradation assay was conducted. The seven-day-old *pru2* mutant and the wild-type seedlings were transferred to an MS or LP medium for 3 d and then harvested for the protein extraction. When HIS–FSD1 or HIS–TCP8 was incubated with the total proteins from the wild-type seedlings grown on an MS or LP medium, the abundance of HIS–FSD1 or HIS–TCP8 showed no obvious differences between MS and LP treatments (Figure 6A,B), indicating that FSD1 and TCP8 were not degraded during Pi starvation, and then the FSD1 and TCP8 were not the targets of PRU2 under LP stress. When HIS–VOZ1 was incubated with the total proteins from the wild-type seedlings grown on an MS or LP medium, the abundance of the HIS–VOZ1 incubation with LP-treated wild-type extracts was reduced more quickly than that with MS-treated wild-type extracts; however, this degradation of HIS–VOZ1 was similar in the *pru2* mutant (Figure 6C), suggesting that VOZ1 was not the target of PRU2 under LP stress. Different from VOZ1, the accumulation of HIS–PHL3 was elevated when incubated with LP-treated wild-type extracts compared to that with MS-treated wild-type extracts (Figure 6D), which is consistent with its related function in response to Pi starvation [31]. No obvious difference was observed, when HIS–PHL3 was incubated with protein extracts of the *pru2* mutant under Pi-sufficient or Pi-deficient conditions (Figure 6D), suggesting that PHL3 was not the target of PRU2 under LP stress.

When HIS–RPL10 and GST–CK2α1 were incubated with the protein extracts from the wild-type plants grown on an LP medium, the accumulation of these two recombinant fusion proteins were distinctly reduced, whereas the accumulations of HIS–RPL10 and GST–CK2α1 were almost not reduced and remained relatively high in the *pru2* mutant grown on an LP medium (Figure 6E,F), indicating that RPL10 and CK2α1 were the targets of PRU2 under LP stress.

### 2.6. Arabidopsis CK2α1 Phosphorylated PHT1;1 at Ser-514

**Figure 7:**
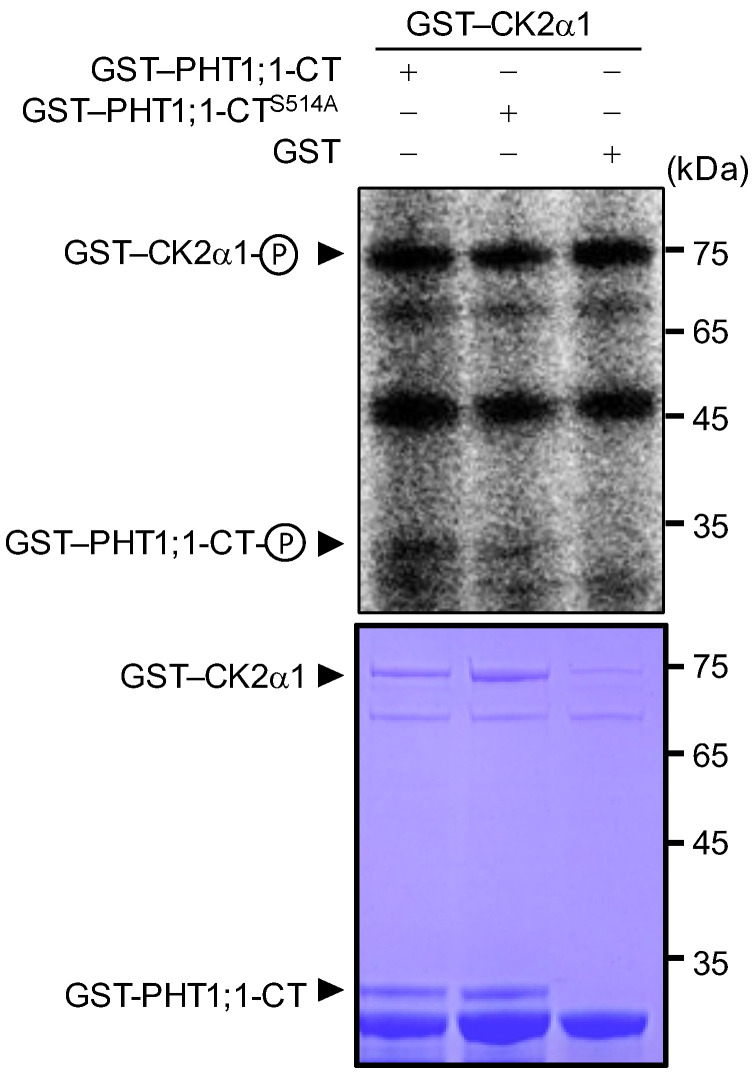
In vitro phosphorylations of GST–PHT1;1–C-terminus(CT), GST–PHT1;1–CT^S514A^, and GST alone by CK2α1 with the respective CBB staining, demonstrating that CK2α1 phosphorylated PHT1-CT in vitro.

Rice PHT1 protein—OsPT8—is a high-affinity Pi transporter and participates in rice Pi uptake and homeostasis [32]. Rice kinase holoenzyme—OsCK2α3/β3—phosphorylates OsPT8 at Ser-517 in the hydrophilic C-terminus (CT) under Pi-sufficient conditions, and this phosphorylation results in OsPT8 retention in the ER [17]. More interestingly, OsCK2β3 is degraded during Pi starvation [17]. As Arabidopsis CK2α1 interacted with PRU2 (Figure 5B,C) and PRU2 modulated the degradation of CK2α1 under LP stress (Figure 6F), we then wondered whether Arabidopsis CK2α1 phosphorylated PHT1;1. In vitro phosphorylation assay was conducted with the recombinant fusion proteins GST–CK2α1 and GST–PHT1;1-CT. As shown in Figure 7, CK2α1 phosphorylated PHT1;1-CT in vitro.

A previous report showed that the phosphorylation of Arabidopsis PHT1;1 at Ser-514 prevents AtPHT1;1 from exiting from the ER to the PM [15]. In addition the Arabidopsis PHT1;1 Ser-514 residue was conserved with OsPT8 Ser-517 [17,33], a site of phosphorylation by OsCK2α3 [17]. To investigate whether Arabidopsis PHT1;1 Ser-514 was a putative phosphorylation site of CK2α1, this residue was mutated to Ala (PHT1;1–CT^S514A^), mimicking the non-phosphorylated PHT1;1–CT. The *in vitro* phosphorylation assay showed that the phosphorylation signal of PHT1;1–CT^S514A^ by CK2α1 was reduced relative to the normal PHT1;1–CT (Figure 7), suggesting that Ser-514 of PHT1;1 maybe a phosphorylation residue of CK2α1.

## 3. Discussion

### 3.1. PRU2 Was a Vital Regulator in Arabidopsis Response to Pi Starvation

Phosphorus is an essential nutrient for plant growth and development, whereas Pi concentration in soil is typically 10 µM or less [1] and plants often suffer from LP stress. Arabidopsis PHT1;1 and PHT1;4 are two major Pi transporters functioning in Arabidopsis Pi acquisition from environments [19]. Arabidopsis PHT1;1 and PHT1;4 are precisely modulated. During Pi starvation, the transcript levels of Arabidopsis *PHT1;1* and *PHT1;4* are up-regulated by several transcription factors, such as PHR1, MYB62, WRKY75, WRKY45, and NIGT1s [25,34,35,36,37]. Arabidopsis PHT1;1 and PHT1;4 are also modulated at the post-translational levels. Arabidopsis PHF1 plays a major role in regulating the trafficking of PHT1;1 from the ER to the PM [14]. In addition, the phosphorylation status of the Ser-514 residue of Arabidopsis PHT1;1 influences its subcellular location; the phosphorylation of Arabidopsis PHT1;1 Ser-514 results in PHT1;1 retention in the ER [15].

**Figure 8:**
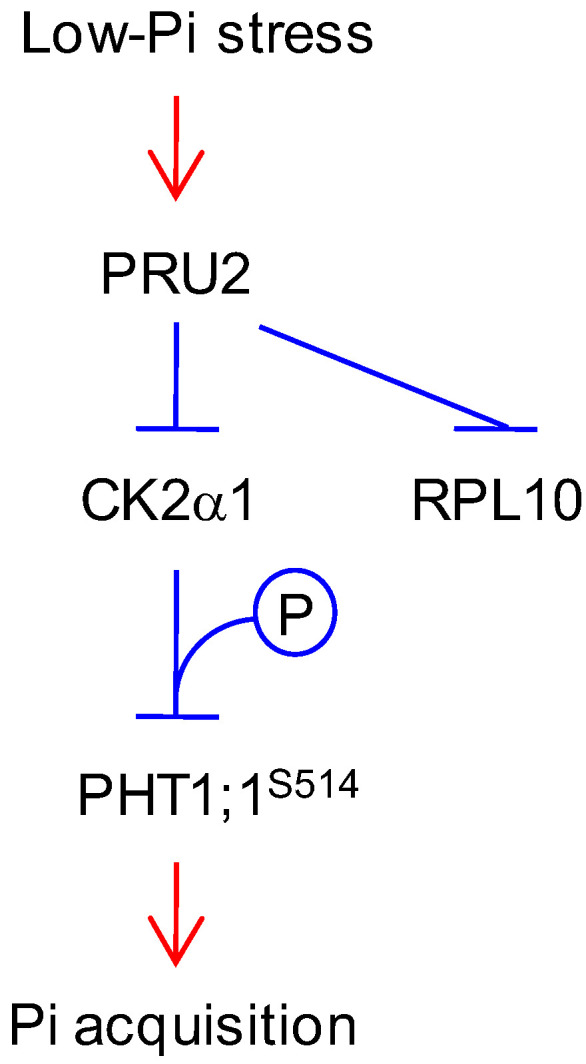
Hypothetical model of PRU2 modulating Pi acquisition under LP stress. Under LP stress, the protein accumulation of PRU2 was elevated; PRU2 interacted with CK2α1 and RPL10 and degraded these two proteins; CK2α1 could not phosphorylate PHT1;1 at Ser-514, which benefited PHT1;1 from exiting from the ER and moving to the PM and then increased Arabidopsis Pi acquisition.

In this study, we assessed over 400 T-DNA insertion mutants of the putative ubiquitin E3 ligase under As(V) stress and obtained a mutant *pru2* which was tolerant to As(V) stress (Figure 1). The *pru2* mutant was defective in Pi uptake, and complementation with *PRU2* restored this to wild-type plants (Figure 1). With further molecular and biochemical assays, we proposed a working model for PRU2 to modulate Pi acquisition (Figure 8). During Pi starvation, the accumulation of PRU2 protein was elevated; PRU2 interacted with a kinase CK2α1 and a ribosomal protein RPL10 and degraded these two proteins. PRU2 degraded the kinase CK2α1, and then, CK2α1 could not phosphorylate PHT1;1 Ser-514. As a result, more PHT1;1 proteins exited from the ER and moved to the PM, which increased Arabidopsis Pi acquisition.

### 3.2. PRU2 Was Involved in a Cross-Talk between the Stress and the Nutrient Deficiency

Previous reports showed that PRU2, also named AtATL78, participates in abiotic stress. The transcript of *AtATL78* is induced by cold stress and repressed by drought stress [29]. The *atatl78* mutant and *AtATL78* RNAi lines show cold-tolerant phenotypes, whereas they were more sensitive to drought stress compared with wild-type plants, suggesting that AtATL78 plays an opposing role in Arabidopsis response to cold and drought stress [29]. Later, AtATL78 was reported to mediate an ABA-dependent stomatal closure during drought stress [38]. Recently, Jiménez-Morales et al. [39] found that the transcript of *AtATL78* is elevated by an insertion of a TATA box within the core promoter region, and this change at the transcription level is associated with Arabidopsis drought tolerance. These data demonstrated that AtATL78 participates in Arabidopsis resistance to drought stress.

In this work, we found that during Pi starvation, AtATL78/PRU2 degraded the protein RPL10 (Figure 6E). RPL10 was associated with transcription factors’ functions [40,41]. In addition, Arabidopsis NIK1, a leucine-rich repeat receptor-like kinase, was a virulence target of the *begomovirus* nuclear shuttle protein (NSP) and led to the global translation suppression and translocation of the downstream component RPL10 to the nucleus [42]. Then, PRU2 may co-regulate Arabidopsis antiviral immunity and adaption to Pi deficiency.

## 4. Materials and Methods

### 4.1. Plant Materials and Growth Conditions

The Col-0 ecotype was used as wild-type *Arabidopsis thaliana* in this study. A T-DNA insertion line Salk_058308C (named as *pru2* in this study) was ordered from ABRC (http://www.arabidopsis.org/abrc).

For the complementation lines, a 2776-bp DNA fragment containing a promoter sequence and the coding region of *PRU2* was cloned into the *pCAMBIA1391* vector, designated as *ProPRU2:PRU2–GFP*. The *ProPRU2:PRU2–GFP* construct was introduced into the *pru2* mutant using the floral-dip method [43], and homozygous single-copy lines were obtained.

For the plant growth, Arabidopsis seeds were surface-sterilized, treated at 4 °C for 72 h and then germinated and grown on an MS medium containing 3% (*w*/*v*) sucrose and 0.8% (*w*/*v*) agar at 22 °C for a 16h daily light period at 80 μmol m^−2^s^−1^ (Philips, Philips Electronics Ltd, Markham, USA; TLD 36W/865 cool daylight), unless otherwise indicated. An LP medium was made by modifying the MS medium to contain 10 μM Pi and agarose (Promega) instead of agar.

For the root acidification assay, roots from seven-day-old plants were grown on an MS or LP medium containing 0.003% (*w*/*v*) bromocresol purple (pH: 6.6), and the changes in the acidity of the medium were visualized.

### 4.2. Pi Content Measurement and ^32^Pi Uptake Assay

Pi contents in genotypes were quantified as described previously [44]. Each experiment was performed in triplicate, and a group of 30 seedlings was used as one biological sample. Three independent experiments were performed.

The ^32^Piuptake assay for plants was conducted as described previously [36].

### 4.3. RT-qPCR Assay

The RT-qPCR assay was conducted as described previously [45], using an SYBR Green PCR Master Mix (Life Technologies) on a 7500 Real Time PCR System (Applied Biosystems). Relative quantitative results were calculated by normalization to *Actin2/8*. Each experiment was performed in biological triplicate, and a group of ~120 seedlings was used as one biological replicate. The primers used are listed in Table S1.

### 4.4. In Vitro Self-Ubiquitination Assay

In vitro self-ubiquitination assays of the MBP–PRU2^78-219^ protein (500 ng) were conducted using anti-MBP antibody (New England BioLabs) for MBP–PRU2^78-219^and anti-FLAG antibody (Medical& Biological Laboratories) for FLAG–Ub,as described previously [46].

### 4.5. Yeast Two-Hybrid Assay

Arabidopsis seven-day-old wild-type seedlings were transferred to an LP medium for 1, 3, 5, and 7 d and then harvested for LP yeast library construction. Briefly, it was constructed by extracting mRNA and cloning the cDNA into the vector *pGADT7* (AD), followed by the transformation of the recombinant vector into the yeast strain Y187. The full-length coding sequence of *PRU2* was cloned into the bait vector *pGBKT7* (BD). The LP library was screened using yeast mating according to the Matchmaker Gold Yeast Two-Hybrid System manufacturer’s protocol (Clontech).

To confirm the protein–protein interactions, the full-length and truncated *VOZ1*, *TCP8*, *RPL10*, *PAF1*, *CAB1*, *FSD1*, and *PHL3* were cloned into the vector *pGADT7* (AD), co-transformed with *pGBKT7-PRU2* into the yeast strain AH109 and plated on an SD/-Leu/-Trp/-His-selective medium (-LWH). To confirm the interaction between PRU2 and CK2αs or CK2βs, the full-length *CK2αs* or *CK2βs* was cloned into the vector *pPR3-N*, co-transformed with *pBT3-SUC-PRU2* into the yeast strain NMY51 and plated on an SD/-Leu/-Trp/-His/-Ade-selective medium (-LWHA). The primers used for the yeast two-hybrid system are list in Table S1.

### 4.6. Protein Expression and Cell-Free Degradation Assay

The deletion derivative of *PRU2* was cloned into the *pMAL-c5x* to generate *MBP–PRU2^78-219^*. The *CK2α1* was cloned into the *pGEX-4T-1* to generate *GST–CK2α1*. The *VOZ1*, *FSD1*, *TCP8*, *RPL10*, and *PHL3* were cloned into the *pET30a* to generate *HIS* fusion constructs. The recombinant constructs were transformed into *E. coli* strain BL21. The *E. coli* cells were induced with 0.5 mM IPTG overnight at 18 °C and collected by centrifugation at 4000 rpm for 15 min. The fusion proteins were purified with glutathione-sepharose, amylose resin, or Ni-sepharose.

A cell-free degradation assay was conducted as described previously [44]. To monitor the degradation of the recombinants, i.e., HIS–FSD1, HIS–TCP8, HIS–VOZ1, HIS–PHL3, HIS–RPL10, and GST–CK2α1, 250 ng of each purified recombinant protein were incubated in 20 µL of the plant protein extract (50 μg) at 22 °C for the indicated time periods. The abundances of the recombinant proteins were analyzed by immunoblotting with an anti-GST or anti-HIS antibody.

### 4.7. In Vitro Pull-Down Assay

The purified GST–CK2α1 or GST protein was incubated with an equal volume of glutathione-sepharose (GE Healthcare) in a binding buffer (200 mM NaCl, 10 mM Na_2_HPO_4_, 1.8 mM KH_2_PO_4_, 2.7 mM KCl, 1 mM PMSF, and 5 mM DTT) at 4 °C for 2 h. The mixture was washed three times with a buffer I (200 mM NaCl, 10 mM Na_2_HPO_4_, 2.7 mM KCl, and 1.8 mM KH_2_PO_4_) and then aliquoted into two equal parts. Equal amounts of the purified MBP and MBP–PRU2^78-219^ were added into the mixture and incubated at 4 °C for 2 h. Then, the mixture was rinsed three times with buffer I, and the bound proteins were boiled in a 1 × SDS loading buffer for 5 min and then examined by immunoblotting using an anti-MBP antibody.

### 4.8. Phosphorylation Assay

The sequence of the hydrophilic CT of *PHT1;1* was fused to *pGEX-4T-1* and resulted in *GST*–*PHT1;1-CT*. The construct *GST*–*PHT1;1-CT^S514A^* was generated from *GST*–*PHT1;1-CT*, using the site-directed mutagenesis technology. The fusion proteins were expressed in *E. coli* BL21 and purified with glutathione-sepharose beads. An in vitro phosphorylation assay was conducted as described previously [33].

### 4.9. Accession Numbers

The sequence data from this paper can be found in the EMBL/GenBank data libraries under the following accession numbers: *PRU2* (AT1G49230), *PHT1;1* (AT5G43350), *PHT1;4* (AT2G38940), *FSD1* (AT4G25100), *VOZ1* (AT1G28520), *CAB1* (AT1G29930), *TCP8* (AT1G58100), *RPL10* (AT1G14320), *PAF1* (AT5G42790), *PHL3* (AT4G13640), *CK2α1* (AT5G67380), *CK2α2* (AT3G50000), *CK2α3* (AT2G23080), *CK2α4* (AT2G23070), *CK2β1* (AT5G47080), *CK2β2* (AT4G17640), *CK2β3* (AT3G60250), *CK2β4* (AT2G44680), *ACT2* (AT3G18780), and *ACT8* (AT1G49240).

## 5. Conclusions

In summary, we identified the function of PRU2 in Arabidopsis response to the LP stress. PRU2 functioned as an ubiquitin E3 ligase, and its protein accumulation was elevated during Pi starvation. The *pru2* mutant showed a reduced Pi uptake capacity relative to the wild-type plants, and the complementation lines rescued its defects, suggesting that PRU2 participated in Pi acquisition. PRU2 interacted with CK2α1 and RPL10 and degraded these two proteins under LP stress. CK2α1 may phosphorylate PHT1;1 at Ser-514, which resulted in PHT1;1 retention in the ER. In addition, the degradation of CK2α1 by PRU2 facilitated the PHT1;1 to exit from the ER to the PM, thus promoting Arabidopsis Pi uptake. Taken together, these data suggested that Pi-starvation-responsive PRU2 is a positive regulator in modulating Pi acquisition in Arabidopsis response to LP stress.

## Figures and Tables

**Table 1:** Putative targets of PRU2 via yeast two-hybrid screening.

Gene	Full Name of the Gene	Gene Number
*VOZ1*	VASCULAR PLANT ONE ZINC FINGER PROTEIN 1	AT1G28520
*FSD1*	FE SUPEROXIDE DISMUTASE 1	AT4G25100
*CAB1*	CHLOROPHYLL A/B BINDING PROTEIN 1	AT1G29930
*TCP8*	TCP DOMAIN PROTEIN 8	AT1G58100
*RPL10*	RIBOSOMAL PROTEIN L10	AT1G14320
*PAF1*	PROTEASOME ALPHA SUBUNIT F1	AT5G42790
*PHL3*	PHR1-LIKE 3	AT4G13640
*CK2* *α* *1*	CASEIN KINASE II, ALPHA CHAIN 1	AT5G67380

## Data Availability

The primers used in this study have been listed in the Table S1.

## Supplementary Materials

The following supporting information can be downloaded at: https://www.mdpi.com/article/10.3390/ijms23042273/s1.
