# Supplementary material for: The Ubiquitin E3 Ligase PRU2 Modulates Phosphate Uptake in Arabidopsis
Source: Int J Mol Sci. 2022 Feb 18;23(4):2273. doi: 10.3390/ijms23042273 (PMC8874776; doi:10.3390/ijms23042273)
Supplement: Supplementary file 1 [file ijms-23-02273-s001.zip › ijms-1533824-supplementary.pdf]

Table S1. Primer sequences used in this study.

Primer Name	Forward Primer (5’ → 3’)	Reverse Primer (5’ → 3’)
For qRT-PCR assay		
Actin2/8	ACGGTAACATTGTGCTCAGTGGTG	CTTGGAGATCCACATCTGCTGGA
PRU2	GGAACGTGTGAAGCTGTTGC	AGTCGGCAATCTTCTCGCAT
PHT1;4	TCAATGGCGTTGCCTTCTGGT	ATCACCAAGCCACCCGAAA
For mutant identification		
pru2	TTGTTTTTAAGTGTAGACCGCTTG	GTCCCTAGAAGCAATTCTGGG
For constructs		
proPRU2:PRU2-GFP	AAGCTTCATAAGTCATCAAAATAGCAATTACC	ACTAGTCCTAAACCAGCGTATCCATCT
MBP-PRU2$^{78-219}$	GTCGACAGATGTGCCTTGAGGTGCTC	GAATTCTCACCTAAACCAGCGTATCCATCTTTC
PRU2-BD	GAATTCATGTGCACTACCATTTCCATTTCC	GTCGACTCACCTAAACCAGCGTATCCATCTTTC
PRU2-pCub	ATTAACAAGGCCATTACGGCCATGTGCACTACCATTTCCATTTCC	AACTGATTGGCCGAGGCGGCCCCCTAAACCAGCGTATCCATCTTTC
VOZ1-AD	GAATTCATGACGGGGAAGCGATC	CCCGGGTCAGGGGATATAATAGTCGCTTAGA
VOZ1$^{96-310}$-AD	ATGGAGGCCAGTGAATTCATGAGGAAGATGATGCTACCAG	TCGAGCTCGATGGATCCCTCACTCACTCTCCAGAACCGTG
FSD1-AD	GAATTCATGGCTGCTTCAAGTGCTG	GGATCCCTTAAGCAGAAGCAGCCTTGG
RPL10-AD	GAATTCATGGGAAGAAGACCTGCG	GGATCCCTCAGTAGTGGGCTGGCAA
TCP8-AD	GAATTCATGGATCTCTCCGACATCC	GGATCCCTCACTCAGAGCTATTTGAGTTCTC
CAB1-AD	ATGGAGGCCAGTGAATTCATGGCCGCCTCAACAAT	TCGAGCTCGATGGATCCCTCACTTTCCGGGAACAAAGT
PHL3-AD	ATGGAGGCCAGTGAATTCATGTACTCGGCGATTCGG	TCGAGCTCGATGGATCCCTCATCCAATGCTACTACTAGGCATA
PAF1-AD	ATGGAGGCCAGTGAATTCATGTTCAGAAACCAATACGACAC	TCGAGCTCGATGGATCCCTTACATTTCCATTGGAGCCA
CK2α1-pNub	ATTAACAAGGCCATTACGGCCATGATAGATACGCTTTTCTTCTTGTTCTT	AACTGATTGGCCGAGGCGGCCTCATTGACTTCTCATTCTGCTGGTTTC
CK2α2-pNub	ATTAACAAGGCCATTACGGCCATGCACCTAATCTTCTTCTTCTCCTA	AACTGATTGGCCGAGGCGGCCCTATTGAGTCCTCATTCTGCTGCTT
CK2α3-pNub	ATTAACAAGGCCATTACGGCCATGTCGAAAGCTAGGGTTTATACAGA	AACTGATTGGCCGAGGCGGCCTTACTGAGTTCGTAGTCTGCTGCTC
CK2α4-pNub	ATTAACAAGGCCATTACGGCCATGGCCTTAAGGCCTTGTACTG	AACTGATTGGCCGAGGCGGCCTCACTGGCTGCGCGGCG
CK2β1-pNub	ATTAACAAGGCCATTACGGCCATGTATAGAGACAGAGGAACGGT	AACTGATTGGCCGAGGCGGCCTCACGGTTTGTGTAATTTGAACCCA
CK2β2-pNub	ATTAACAAGGCCATTACGGCCATGTATAGGGAGAGAGGTATGGTTG	AACTGATTGGCCGAGGCGGCCTCACGGCTTGTGTAGCTTGAACC
CK2β3-pNub	ATTAACAAGGCCATTACGGCCATGTACAAGGAACGTAGTGGAGG	AACTGATTGGCCGAGGCGGCCTCATGGTTTGTGTACCTTGAAGCCA
CK2β4-pNub	ATTAACAAGGCCATTACGGCCATGTACAAGGATCGGAGTGGAG	AACTGATTGGCCGAGGCGGCCTCATTGTTTGTTGTGTACCTTAAAGCCA
GST-CK2α1	GAATTCATGATAGATACGCTTTTCTTCTTGTTCT	GTCGACTCATTGACTTCTCATTCTGCTGG
HIS-FSD1	GAATTCATGGCTGCTTCAAGTGCTG	GTCGACTTAAGCAGAAGCAGCCTTGG
HIS-TCP8	GAATTCATGGATCTCTCCGACATCC	AAGCTTTCACTCAGAGCTATTTGAGTTCTC
HIS-VOZ1	GAATTCATGACGGGGAAGCGATC	GTCGACTCAGGGGATATAATAGTCGCTTAGA
HIS-PHL3	GAATTCATGTACTCGGCGATTCGG	GTCGACTCATCCAATG CTACTACTAGGCATA
HIS-RPL10	GAATTCATGGGAAGAAGACCTGCG	GTCGACTCAGTAGTGG GCTGGCAA
